# Spinal Cord Injury and Loss of Cortical Inhibition

**DOI:** 10.3390/ijms23105622

**Published:** 2022-05-17

**Authors:** Bruno Benedetti, Annika Weidenhammer, Maximilian Reisinger, Sebastien Couillard-Despres

**Affiliations:** 1 Institute of Experimental Neuroregeneration, Paracelsus Medical University, 5020 Salzburg, Austria; bruno.benedetti@pmu.ac.at (B.B.); annika.weidenhammer@meduniwien.ac.at (A.W.); m.reisinger@stud.pmu.ac.at (M.R.); 2 Spinal Cord Injury and Tissue Regeneration Center Salzburg (SCI-TReCS), 5020 Salzburg, Austria; 3 Austrian Cluster for Tissue Regeneration, 1200 Vienna, Austria

**Keywords:** cortical inhibition, spinal cord injury, neocortex, disinhibition, interneuron, transcranial magnetic stimulation, TMS

## Abstract

After spinal cord injury (SCI), the destruction of spinal parenchyma causes permanent deficits in motor functions, which correlates with the severity and location of the lesion. Despite being disconnected from their targets, most cortical motor neurons survive the acute phase of SCI, and these neurons can therefore be a resource for functional recovery, provided that they are properly reconnected and retuned to a physiological state. However, inappropriate re-integration of cortical neurons or aberrant activity of corticospinal networks may worsen the long-term outcomes of SCI. In this review, we revisit recent studies addressing the relation between cortical disinhibition and functional recovery after SCI. Evidence suggests that cortical disinhibition can be either beneficial or detrimental in a context-dependent manner. A careful examination of clinical data helps to resolve apparent paradoxes and explain the heterogeneity of treatment outcomes. Additionally, evidence gained from SCI animal models indicates probable mechanisms mediating cortical disinhibition. Understanding the mechanisms and dynamics of cortical disinhibition is a prerequisite to improve current interventions through targeted pharmacological and/or rehabilitative interventions following SCI.

## 1. Introduction: SCI Harms the Brain

Traumatic SCI is a sudden and unpredictable incident that destroys portions of the spinal cord, leading to motor and sensory deficits, as well as dysfunctions of the somatic and autonomic nervous systems [1]. Beyond the loss of movement control, typical deficits include the loss of bladder and bowel control, declined sexual functions and chronic pain, among others [1,2]. SCI can occur at any age, and the damage is irreversible. However, constant improvements in healthcare and treatment, as well as increased awareness about the needs of patients over the last century, have significantly ameliorated the quality of life and lifespan following SCI [3,4,5,6,7,8,9]. Thus, it is even more pressing to identify interventions enabling the recovery of functions lost after SCI. The recovery of muscle control is a crucial element to improve the quality of life and the autonomy of SCI patients. Accordingly, rehabilitation and active lifestyle have been recognized as crucial processes that help to regain independence and to reduce health complications resulting from prolonged inactivity [10,11,12,13,14]. Nevertheless, the timely implementation of efficient strategies remains often neglected, affecting motor recovery and, together with accompanying morbidities, decreasing the likelihood of returning to a fully independent life routine [15,16]. Furthermore, various therapies addressing the symptoms of SCI are being developed with promising outcomes for management and reduction in secondary damage, increased neuroprotection and improved neuroregeneration [5,17]. However, despite constant improvements, an effective cure, leading to major functional recovery based on the regeneration of neuronal connectivity across the lesion, is still missing [17,18,19,20].

Many of the neurons that become disconnected following SCI reside outside the spinal cord, such as the motor neurons of the primary motor cortex, which are crucial for the control of voluntary movements. These disconnected neurons are a resource for the long-term regeneration and functional recovery of the central nervous system. However, the axotomy resulting from SCI has an impact on the physiology of the cortical and corticospinal network [21,22,23], which can complicate or even hinder the recovery process. Several attempts to address the clinical symptoms of SCI have therefore explored the possibility to retune neuronal activity in the corticospinal network [24,25]. Finding ways to reconnect cortical motor neurons to their original targets constitutes a daunting task. In addition, early assessments of the severity of SCI, especially in an acute situation, are difficult and inherently inaccurate [26]. This lack of knowledge is a major hurdle for the design of an effective and patient-specific treatment. Therefore, the assessment of cortical activity in SCI patients, for example, using electro-encephalography (EEG), transcranial magnetic stimulation (TMS), etc., has been extremely convenient, as it relies on non-invasive techniques [27,28]. In addition, animal models are available to resolve the molecular mechanisms of brain dysfunction after SCI. In this review, we take advantage of the complementarity of clinical research and basic research to offer a multifaceted overview about cortical network dysfunction after SCI and putative underlying mechanisms.

## 2. TMS as a Method to Analyze the Loss of Inhibition after SCI

Following SCI, extensive functional plasticity and the reorganization of neuronal circuits often involve altered inhibitory neurotransmission [29,30,31,32,33,34,35,36,37]. Although brain disinhibition has been explored extensively for pathologies such as peripheral deafferentation and stroke [38,39,40,41,42,43], it remains resolved to a lesser extent in the context of SCI [44]. The establishment of direct comparisons between cortical output and motor performance after SCI and after other pathologies, such as peripheral deafferentation or stroke, is hindered by discrepancies intrinsic to various conditions. Therefore, this review focuses on the phenomenon of cortical disinhibition as a central component of the pathophysiology of SCI. Even under such focused premises, disinhibition appears as a startlingly heterogeneous process that needs to be first disentangled to be considered in the design of therapy and treatment following SCI.

**Figure 1:**
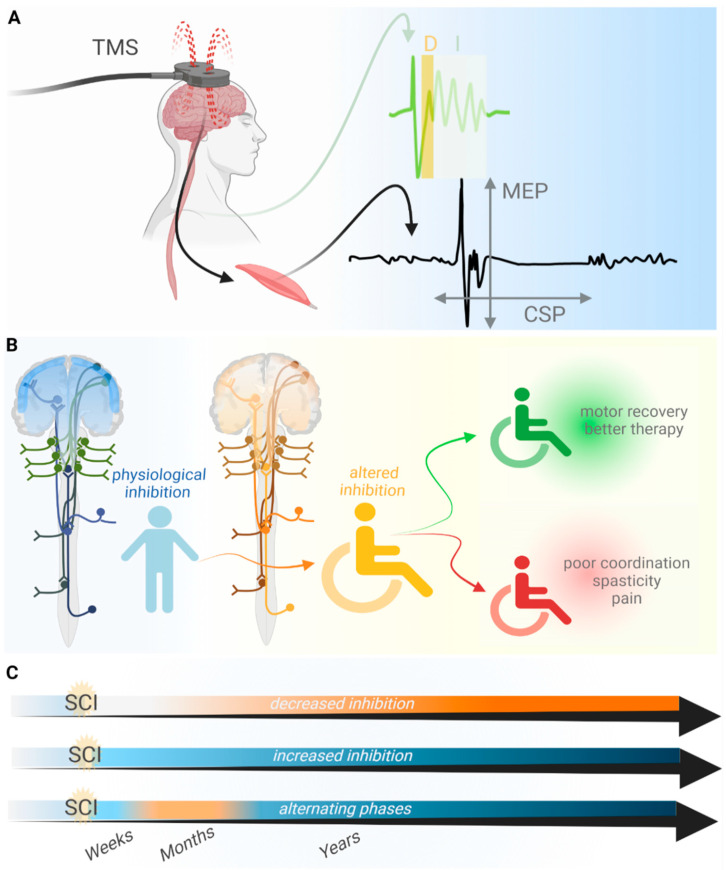
TMS enables measurement of cortical and subcortical excitability and inhibition after SCI. (**A**) TMS-evoked activity reveals the direct and indirect trans-synaptic activation of corticospinal neurons evident as D- and I-waves in descending volleys from the spinal cord. Changes in I-waves reflect altered cortical inhibition after SCI. Similarly, changes in motor evoked potentials (MEP) and cortical silent period (CSP), evoked by TMS pulses and recorded with electromyography, reflect altered integrity of the corticospinal tract after SCI. (**B**) The physiological balance (blue) between excitation and inhibition in cortical and corticospinal networks is perturbed (yellow) by SCI. On one hand, altered inhibition supports rewiring and motor recovery and appears as an exploitable condition in therapeutic treatments. On the other hand, altered inhibition contributes to detrimental aspects, such as exacerbated pain, spasticity and poor motor coordination. (**C**) Altered balance between excitability and inhibition of cortical and corticospinal areas is long-lasting and can endure for decades after SCI. However, alterations vary qualitatively between patients, and therefore, inhibition may be decreased [2,34,57,58,59,60,61,62] or increased [50,63,64] as a consequence of SCI. Moreover, there may be phases of decreased excitability, weeks and years after SCI, interspersed with transiently decreased inhibition months after SCI [65]. Such transient events can contribute to fluctuation in the balance of excitation and inhibition over time during the recovery process. Better resolution of the pathophysiological mechanisms of altered inhibition can allow the determination of relevant factors for the occurrence, duration, heterogeneity and alternation of such phases. This figure was created with biorender.com. Adapted from “Ascending and Descending Spinal Pathways”, by biorender.com. Retrieved from https://app.biorender.com/biorender-templates (accessed on 29 March 2022).

Here, we consider the specific patterns of functional cortical derangement after SCI, focusing primarily on works based on TMS. TMS is a technique that evokes neuronal activity by the juxtaposition of magnetic coils to the skull and the generation of magnetic fields, delivered as pulses, in selected and focalized cortical areas. Therewith, evoked electrophysiological activity can be measured from the cortex, spinal cord and muscles targeted by the stimulated motor areas, and specific parameters in the readout reflect the relative excitability and inhibition of cortical and corticospinal circuits [45,46]. However, TMS has limitations, including the poor resolution of direct and indirect stimulus effects, ambiguities in the causal relationship of therapeutic TMS and behavioral improvement, and caveats when combining TMS with some other analytical techniques [47,48]. Most relevantly, the reliability of TMS metrics varies depending on the residual muscle strength of the tested muscle, which, if not accounted for, can significantly affect measurements assessing recovery from SCI [49]. Nevertheless, TMS is useful to stimulate the brain safely and non-invasively in awake patients and to measure specific brain activities and functional alterations after SCI [50,51,52,53]. Therefore, decades of work based on TMS have successfully outlined the traits of altered brain physiology after SCI (Figure 1) [51,54,55,56].

## 3. Loss of Inhibition Promotes Motor Recovery after SCI

Early works have suggested that the alteration of inhibitory mechanisms ensues from SCI and causes increased excitability and plasticity in the central nervous system [34,58]. Accordingly, reduced thresholds for effective TMS of muscles innervated by segments above the lesion site were measured in patients with complete thoracic SCI, and this observation was interpreted as evidence for hyper-excitability and remodeling after injury [59]. The loss of inhibition and network remodeling was also suggested by experiments involving paired-pulse TMS [60]. Namely, increased Indirect-wave (I-wave) facilitation was detected after SCI, whereas the motor conduction time remained unaltered. In this context, I-waves originate from the TMS-evoked output of cortical excitatory neurons, which are regulated by a network of GABAergic interneurons [66,67]. Thus, it was proposed that increased I-wave facilitation after SCI results from a loss of cortical inhibition. The modulation of I-waves resulting from dynamic interaction of the excitatory and inhibitory network is relevant to determine the extent of spinal motor neuron activation [68,69,70,71]. In this scenario, cortical hyper-excitability resulting from disinhibition may therefore strengthen the output of spared fibers during motor recovery. Thus, the loss of inhibition can, in this view, ameliorate the cortical output, strengthen motor control and improve motor recovery [72].

Independent evidence converging towards a similar conclusion, i.e., the occurrence of the loss of inhibition after SCI, was obtained by measuring cortical silent periods (CSP). A CSP indicates the duration of the transient decrease in motor neuron excitability after a TMS pulse. SCI was found to shorten the CSP [57], which can be regarded as a consequence of disinhibition and a contribution of cortical remodeling to facilitate motor performance recovery [57,73]. In line with these studies, a clinical study supporting cortical remodeling and disinhibition after SCI has shown that the peripheral stimulation of lower limbs can prime contextual hand flection in SCI patients [44]. A more recent work agrees with the occurrence of disinhibition after SCI [61]. In this case, the experimental readout was based on measurements of the resting motor threshold and motor evoked potentials (MEP) upon TMS. Thereby, Nardone and colleagues observed that the input/output ratio (i.e., stimulation intensity/MEP) decreased in individuals with SCI, whereas the resting motor threshold remained constant. Based on previous works, the authors have discussed that the combined readout associates with increased excitability of spinal rather than cortical areas [66,74,75,76,77,78,79]. The observed alteration in motor output is therefore caused by the remodeling and loss of inhibition, causing the activation of a larger fraction of the pool of excitatory synapses for a given stimulus intensity. Once more, disinhibition is portrayed as a rescuing effect contributing to motor recovery.

## 4. The Loss of Inhibition Aggravates Symptoms after SCI

The works cited so far suggest that disinhibition and increased CNS excitability are beneficial for motor recovery after SCI. However, there is evidence to suggest that cortical disinhibition is detrimental: the exacerbated loss of cortical inhibition and hyper-excitability can lead to maladaptive plasticity and neuropathic pain [80,81]. For example, suppressed inhibition and damage to the thalamocortical network can lead to neuropathic pain after SCI [2]. Additional manifestations associated with the loss of central nervous system inhibition after SCI are “referred phantom sensations” [62]. In this context, repetitive TMS was used to treat referred phantom sensations, and it was proposed to restore cortical inhibition through the reactivation of silent intracortical interneurons. Moreover, the authors studying reduced short-interval intracortical inhibition after SCI argued in favor of an inhibitory impairment. The latter contributes to defective corticospinal control, hindering the accurate planning and/or termination of voluntary muscle contractions, therefore causing poor motor control [82,83].

In summary, a complex clinical scenario reveals that the loss of inhibition following SCI can be helpful on one side to intensify the output signal of cortical motor neurons to the spinal cord. On the other hand, excessive disinhibition can also contribute to the development of chronic pain and maladaptive plasticity, and it can hinder motor control [81]. Since disinhibition seems to have both pros and cons, it should be considered whether a treatment that alters the degree of cortical inhibition would rather cause harm or benefit. A better resolution over the mechanisms and temporal patterns of disinhibition can be crucial for personalized choices on therapeutic options.

## 5. Alternative Mechanisms and Explanations for Imbalanced Cortical Excitability

Even though the loss of CNS inhibition is often observed after SCI, it may not be a condition that applies to all patients [64] nor fully account for CNS plasticity. Indeed, some reports describing increased CNS excitability after SCI did not conclude a loss of inhibition and therefore did not consider retuning the inhibitory network as a putative therapeutic strategy [52,59,84]. For instance, paired stimulation of peripheral nerve and cortical areas promotes corticospinal transmission through a form of plasticity which does not involve the modulation of inhibitory networks [85]. Moreover, paired associative stimulation has been shown to improve motor recovery [86] and is associated with long-term potentiation of excitatory neurotransmission [86,87] that has been suggested to involve the structural reorganization of the corticomotoneuronal synapses of the cervical spinal cord. Finally, TMS after SCI has been reported to directly increase glutamate receptor activation and strengthen excitatory neurotransmission [88]. Therefore, increased excitatory neurotransmission may also be possible independently of processes of disinhibition.

As a consequence, although not always present, disinhibition may well occur concomitantly with other plasticity mechanisms, resulting in apparent heterogeneity and discrepancy between experimental outcomes [50,63,89]. Furthermore, some works have provided evidence against cortical disinhibition and in favor of increased corticospinal inhibition when using paired-pulse TMS and comparing SCI patients to healthy subjects [50,63]. Moreover, a recent work relying on paired-pulse TMS has measured a specific alteration of MEP amplitude and kinetics that is best explained by diminished capacity to sum up descending excitatory volleys after SCI, rather than by cortical disinhibition [69]. In particular, the authors argue that the most parsimonious explanation for their data was that early corticospinal volleys are insufficient to bring spinal motoneurons to the threshold and that such deficiency is compensated by the later volley. On the other hand, the type of alteration in MEP amplitude and kinetics that was observed in the study was not in line with the expected effects of cortical disinhibition [70,90].

A further example of conflicting outcomes on the matter of cortical disinhibition involves two works based on TMS related to patients with cervical SCI [57,64]. Whereas the first study reports the loss of cortical inhibition as a consequence of SCI [57], the second reveals just the opposite [64]. Remarkably, the second study by Freund and co-workers combines multiple analytical approaches and a larger sample size, resulting in compelling evidence that directly correlates CSP duration to the extent of spinal cord atrophy. It is somehow puzzling to find divergent results in similar studies. However, several factors, including the type of injury, comorbidities, treatments, age and duration of injury, as well as patient demography, motivation and access to support, influence the process of recovery [91], which may contribute to heterogeneity in the course of pathophysiological remodeling of the central nervous system. In this regard, it should also be considered that, besides inter-patient variability, clinical studies on altered excitability and inhibition after SCI are affected by limited accessibility to patients and a lack of options for repeated analyses covering different stages post-injury. Hence, most works focus on few measurements during the chronic phase of SCI (ranging from years to decades after SCI), which is the period providing the highest accessibility from a patient-management perspective. However, it may be revealing to consider what happens to the central nervous system in the very dynamic period shortly after injury, evolving towards the chronic phase [92]. A remarkable study in such a regard analyzes the cortical plasticity of patients from the early weeks post-injury up to a few years after SCI [65]. Interestingly, the study reveals that decreased excitability manifests early (weeks) and lasts for a long time (years) after SCI. However, cortical inhibition is transiently decreased some months after SCI. Hence, an altered balance between excitability and inhibition is the result of alternating events after SCI, and the authors propose that transient disinhibition may be critical for recovery. The unique and novel nature of this work prompts cautious interpretations. Nevertheless, transient loss of inhibition appears once more to be proposed as relevant for motor recovery, as in other earlier studies. Moreover, alternations of phases may explain discrepancies among other works.

Taken together, studies describing altered balances between cortical excitation and inhibition point towards a relevance of this phenomenon in the course of recovery following SCI. However, many confounding factors need to be addressed to improve the comparability of studies made at different clinical centers. Importantly, the severity and position of injury, as well as the time post-injury, need to be considered in the interpretation of TMS outcomes. Moreover, each patient receives a personalized regimen of pharmacological and rehabilitative treatments that has an impact on their cortical plasticity. Therefore, it is necessary for the field to agree on standardized stimulation protocols and methods of analysis that can enable studies with a larger number of patients and adequate controls.

## 6. Possible Causes for the Loss of Inhibition

**Figure 2:**
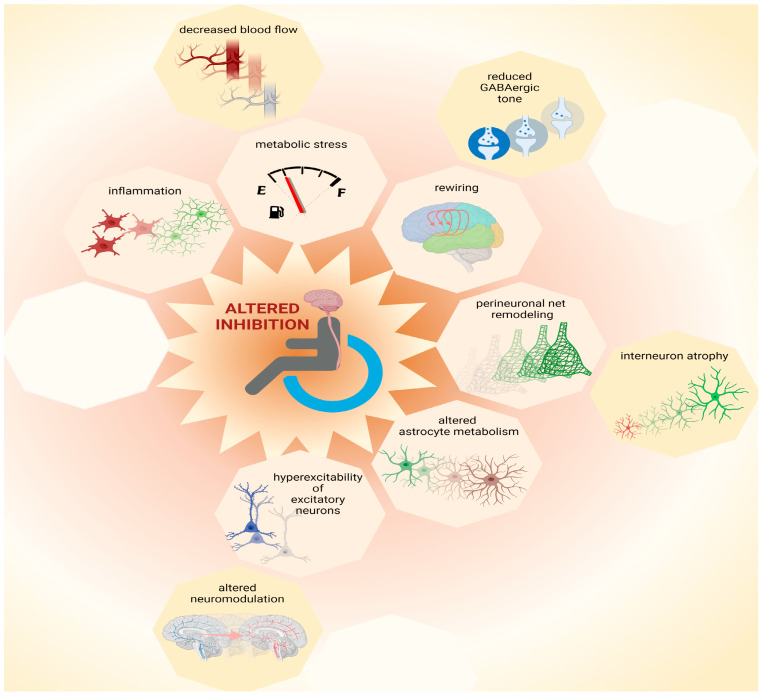
Possible causes for loss of inhibition. Multiple mechanisms contribute to network plasticity and altered inhibition in cortical and subcortical areas after SCI. These include metabolic stress, which can be exacerbated by inflammation and by decreased blood flow, as well as altered astrocytic metabolism. Furthermore, increased excitability of principal (excitatory) neurons may be exacerbated by altered neuromodulation, contributing to increased output volume in corticospinal circuits. Moreover, disinhibition is associated with decreased GABAergic tone that contributes to plasticity during network rewiring. Additionally, remodeling of perineuronal nets, which involves atrophy of interneurons, can contribute to complex patterns of altered inhibition in the central nervous system after SCI. Thus, multiple mechanisms, some of which are yet to be identified (represented by empty octagons) may coexist and combine heterogeneously amongst each other and/or to other pathophysiological components, increasing inter-patient variability. This figure was created with biorender.com.

Pre-clinical research helps to define mechanisms that can converge to an altered ratio of excitation and inhibition following SCI and to refine hypotheses about the reasons for phenotypic heterogeneity, as reported in clinical findings. Focusing on molecular “bottlenecks” of inhibitory neurotransmission, the likely mechanisms of cortical disinhibition after SCI are presented in the following section (Figure 2).

### 6.1. Metabolic Stress

The synthesis of neurotransmitters, such as glutamate and GABA, demand considerable energy consumption [93]. Of these, GABAergic neurotransmission has the highest demand. For this reason, inhibitory neurons such as parvalbumin-positive and axo-axonic interneurons are vulnerable and respond to stress with decreased GABAergic neurotransmission [94]. After SCI, the axotomy of corticospinal neurons may cause stress, spreading from the spinal cord to cortical areas [21,22,23]. Additional sources of stress may be inflammation [95] and impaired cerebral blood flow [96]. Furthermore, stress may coincide with high metabolic demand due to increased cortical excitability [22]. Hence, multiple stressors may affect interneurons, as also suggested by the direct observation of their atrophy after SCI [32]. The alteration in brain network performance as a consequence of dysfunctional interneurons may impact cognition negatively [97,98], such as occasionally documented in SCI patients [99].

### 6.2. Inflammation

Microglia activation plays a pivotal role in SCI. Furthermore, chronic inflammation seems to affect supraspinal regions as well [100]. This is not surprising, given the axotomy of several cortical and subcortical neurons [21,22,23] and the continuity of the spinal cord and brain. Regrettably, although the wave of inflammation after SCI has been well time-resolved near the lesion site [101,102,103], little is known about the spatial distribution of immune cell activation across the CNS following various types of injury and treatment. Thus, it is hard to define which neurons are most exposed to inflammatory processes and which are spared and exclusively undergo plasticity processes independent of inflammation. Among several disinhibitory effects [104], activated microglia affect the activity of proteins that transport chloride across the neuronal cell membrane and cause the dissipation of chloride trans-membrane gradients with consequent reductions in the GABAergic inhibitory drive [105]. Decreased GABAergic drive causes the neurotransmitter GABA to be intrinsically less effective in hyperpolarizing the neuronal membrane to mediate inhibition. The dissipation of chloride gradients upon microglia activation after SCI may therefore contribute to disinhibition as well, documented in other pathologies [105,106].

### 6.3. Remodeling of Perineuronal Nets

The extracellular elements of proteoglycans, known as perineuronal nets, mediate stability in network architecture as well as protection from stress [107]. Dismantling perineuronal nets promotes plasticity and network remodeling [108], which are pertinent for recovery after SCI [109,110]. Furthermore, perineuronal nets are closely associated with specific categories of cortical interneurons [111,112]. Thereby, these extracellular elements can control neuronal synaptic connectivity and intrinsic firing properties [112,113]. Thus, the dismantling of perineuronal nets can affect the activity and connectivity of interneurons, determining altered cortical inhibition after SCI. Strikingly, the advantage of increased plasticity derived from the dismantling of perineuronal nets comes along with the challenge presented by increased metabolic stress. Indeed, perineuronal nets limit excitotoxicity by sheltering synaptic contacts and reducing oxidative stress to which interneurons are most vulnerable [94,114,115]. Since neocortical perineuronal nets undergo remodeling after SCI that is associated with the atrophy of interneurons [32], these extracellular elements may be involved in both beneficial and detrimental aspects of cortical disinhibition.

### 6.4. Altered Astrocyte Metabolism and Physiology

Axotomy and neuronal trauma perturb the physiology of astrocytes [116]. Trauma suffered by cortical principal neurons upon axotomy [21,22,23] may therefore alter signaling from astrocytes to surrounding neurons. Astrocytes can release neuromodulators controlling neuronal activity by gliotransmission [117,118,119]. Moreover, astrocytes are crucial for the metabolism of neurotransmitters [120,121]. Thus, under pathological conditions, altered astrocytic activity may affect neurotransmission, and in particular GABAergic inhibition, via altered metabolic support as well [122]. The involvement of astrocytes in SCI-derived pathology has been widely studied [123], albeit not in cerebral regions.

### 6.5. Rewiring of Cortical Circuits

A physiological process closely tied to disinhibitory mechanisms is the rewiring of cortical circuits after SCI. Like other injuries involving deafferentation of the central nervous system, SCI involves the rewiring of cortical and subcortical areas, as well as the shift of somatotopic representations and changes in competence of motor areas, which relay on significant events of network plasticity [124]. Decreases in GABAergic inhibition appear crucial for network remodeling [125,126] because plasticity and learning are tightly related to changes in cortical GABA [127]. For instance, reduced GABAergic inhibition causes qualitative and quantitative changes in the inducibility of long term potentiation in the neocortex [128,129]. Furthermore, reduced GABAergic tone, i.e., diminished concentrations of extrasynaptic GABA, supports motor plasticity and motor recovery in various types of pathology [126,130]. Thus, several mechanisms that contribute to reduced GABAergic inhibition in the brain after SCI may also partake into the process of cortical rewiring and recovery.

### 6.6. Hyperexcitability of Excitatory Neurons

Some causes of hyperexcitability may be independent from disinhibition. Nevertheless, solving pathological hyperexcitability may necessitate retuning inhibitory components. For instance, axotomy results in the depolarization and hyperexcitability of cortical principal neurons after SCI [22]. The retuning of hyperexcitable neurons may benefit from increased inhibition, for instance, through GABA_B_ receptor signaling, which controls dendritic excitability via intracellular calcium signaling [131]. In contrast, disinhibition has additive exacerbating effects on the intrinsic hyperexcitability of principal neurons. Furthermore, other types of neuromodulation that exert inhibitory control on CNS neurons, e.g., the ones mediated by serotonin, are impaired after SCI [132]. The activation of serotonin receptors has been directly shown to control the excitability of cortical neurons [133]. Additionally, the enrichment of serotonin receptors at the axonal hillock of layer V pyramidal neurons implies a crucial role in the control of cortical functional output [134]. Thus, non-GABAergic neuromodulation can directly control the excitability of principal neurons as well as the excitability of interneurons [135,136]. Since altered serotoninergic neuromodulation can affect directly and indirectly the volume of corticospinal output, serotonin neuromodulation may be a key component in controlling cortical output after SCI. Strikingly, controlling serotoninergic neuromodulation has already proven to support better recovery after SCI [137,138,139].

## 7. Can Therapy Rely on the Loss of Inhibition?

Since disinhibition is integral to plastic rewiring in the central nervous system [124,125,126] and supports motor recovery in pathophysiological conditions [130], it is tempting to hypothesize that therapy after SCI may benefit from actively modulating the balance of excitation and inhibition in cortical and corticospinal networks. Indeed, efforts to improve interventions against SCI have provided encouraging breakthroughs and better understandings of the interconnection between brain plasticity and motor recovery involving the phenomenon of cortical disinhibition.

The repetition of TMS pulses, delivered to the motor cortex at short intervals, modulate corticospinal excitability and improve motor functions, defined as hand movement and dexterity, in incomplete chronic cervical SCI patients [83,140]. The chosen pattern of repetitive stimulation mimicks the pattern of descending late I-waves and reproduces the success of earlier work on incomplete chronic SCI patients [72]. The beneficial effects of such TMS protocols are reported as relying on the modulation of cortical or corticospinal inhibition. Furthermore, TMS protocols of paired associative stimulation are also able to improve corticospinal excitability and provide transient improvements of the motor performance of chronic incomplete cervical SCI patients [141]. In view of these results, the authors argue that the underlying mechanisms rely mainly on processes of long-term potentiation, but a short-term reduction in inhibition facilitates the long-term effects. Regrettably, some studies (e.g., [142]) could not corroborate the effectiveness of repetitive TMS treatments. The authors could not rule out that methodological issues or inter-patient variability contributed to the lack of conclusiveness in their evidence. However, critical differences between studies may also justify different degrees of success. For instance, Kuppuswamy and colleagues treated SCI patients with more heterogeneous types of injury in comparison to the other aforementioned works. Furthermore, Kuppuswamy’s patient medications involved several drugs that affect central nervous system excitability, including an agonist for GABAergic receptors, whereas in more recent work, the administration of drugs known to interfere with cortical excitability was discontinued before the study [141].

Besides TMS-based therapy, periods of loss of cortical inhibition may be useful windows of opportunity for improving recovery based on increased motor exercises. Essentially, lowered interneuron activity in the cortical circuit may, in some SCI patients, become an asset to boost the outcomes of therapy aiming for motor recovery. Following this idea, robotic training successfully achieved increased smoothness and improved aim of movements in chronic cervical SCI tetraplegic patients who had no likelihood of further movement recovery [143]. In such circumstances, motor improvement was monitored with TMS to assess potential changes in the excitability of the central nervous system. Although no direct causative evidence could be provided, the authors explained the effect of the therapy as a modulation of the ratio between excitation and inhibition at the cortical or subcortical level, based on earlier reports on GABA and network reorganization [144].

## 8. Conclusions

Amongst the consequences of SCI on the central nervous system, the loss of inhibition is a common finding, albeit not always observed, and it is likely to fluctuate over time. Changes in cortical excitability involve a plethora of mechanisms, which individual effects may combine in complex and variable outcomes. When reported, the loss of inhibition is mostly proposed to improve motor recovery and can therefore be exploited for that purpose. On the other hand, the loss of inhibition can under some conditions aggravate SCI symptoms in some patients. Thus, the extent of alteration in the balance of excitatory and inhibitory networks should be determined on a patient-to-patient basis and at different times after injury for optimal therapy design. However, this first requires the standardization of assessment methods and the resolution of molecular pathways causing altered cortical dysfunctions in patients after SCI.

## Data Availability

Not applicable.

## References

[1] Ahuja C.S., Wilson J.R., Nori S., Kotter M.R.N., Curt A., Fehlings M.G. (2017). Traumatic spinal cord injury. Nat. Rev..

[2] Masri R., Keller A. (2012). Chronic Pain Following Spinal Cord Injury. Adv. Exp. Med. Biol..

[3] Lifshutz J., Colohan A. (2004). A brief history of therapy for traumatic spinal cord injury. Neurosurg. Focus.

[4] Hulsebosch C.E. (2002). Recent advances in pathophysiology and treatment of spinal cord injury. Am. J. Physiol. Adv. Physiol. Educ..

[5] Cadotte D.W., Fehlings M.G. (2011). Spinal cord injury: A systematic review of current treatment options. Clin. Orthop. Relat. Res..

[6] Venkatesh K. (2019). Spinal cord injury: Pathophysiology, treatment strategies, associated challengeenges. Cell Tissue Res..

[7] Clayton K.S., Chubon R.A. (1994). Factors associated with the quality of life of long-term spinal cord injured persons. Arch. Phys. Med. Rehabil..

[8] Krause J., Saunders L. (2010). Risk of mortality and life expectancy after spinal cord injury: The role of health behaviors and participation. Top. Spinal Cord Inj. Rehabil..

[9] Anneken V., Hanssen-Doose A., Hirschfeld S., Scheuer T., Thietje R. (2010). Influence of physical exercise on quality of life in individuals with spinal cord injury. Spinal Cord.

[10] Van Der Woude L.H.V., De Groot S., Postema K., Bussmann J.B.J., Janssen T.W.J., Post M.W.M. (2013). Active LifestyLe Rehabilitation Interventions in aging Spinal Cord injury (ALLRISC): A multicentre research program. Disabil. Rehabil..

[11] Van den Akker L.E., Holla J.F.M., Dadema T., Visser B., Valent L.J., de Groot S., Dallinga J.M., Deutekom M. (2020). Determinants of physical activity in wheelchair users with spinal cord injury or lower limb amputation: Perspectives of rehabilitation professionals and wheelchair users. Disabil. Rehabil..

[12] Nas K., Yazmalar L., Şah V., Aydin A., Öneş K. (2015). Rehabilitation of spinal cord injuries. World J. Orthop..

[13] Behrman A.L., Harkema S.J. (2007). Physical Rehabilitation as an Agent for Recovery After Spinal Cord Injury. Phys. Med. Rehabil. Clin. N. Am..

[14] Spooren A.I.F., Janssen-Potten Y.J.M., Kerckhofs E., Seelen H.A.M. (2009). Outcome of motor training programmes on arm and hand functioning in patients with cervical spinal cord injury according to different levels of the ICF: A systematic review. J. Rehabil. Med..

[15] Montesinos-Magraner L., Serra-Ano P., García-Masso X., Ramírez-Garcerán L., González L.M., González-Viejo M. (2018). Comorbidity and physical activity in people with paraplegia: A descriptive cross-sectional study. Spinal Cord.

[16] Floríndez L.I., Carlson M.E., Pyatak E., Blanchard J., Cogan A.M., Sleight A.G., Hill V., Diaz J., Blanche E., Garber S.L. (2020). A qualitative analysis of pressure injury development among medically underserved adults with spinal cord injury. Disabil. Rehabil..

[17] Griffin J.M., Bradke F. (2020). Therapeutic repair for spinal cord injury: Combinatory approaches to address a multifaceted problem. EMBO Mol. Med..

[18] Huang H., Sharma H.S., Chen L., Otom A., Al Zoubi Z.M., Saberi H., Muresanu D.F., He X. (2018). Review of clinical neurorestorative strategies for spinal cord injury: Exploring history and latest progresses. J. Neurorestoratology.

[19] Shah M., Peterson C., Yilmaz E., Halalmeh D.R., Moisi M. (2020). Current advancements in the management of spinal cord injury: A comprehensive review of literature. Surg. Neurol. Int..

[20] Hutson T.H., Di Giovanni S. (2019). The translational landscape in spinal cord injury: Focus on neuroplasticity and regeneration. Nat. Rev. Neurol..

[21] Hains B.C., Black J., Waxman S.G. (2003). Primary cortical motor neurons undergo apoptosis after axotomizing spinal cord injury. J. Comp. Neurol..

[22] Nagendran T., Larsen R.S., Bigler R.L., Frost S.B., Philpot B.D., Nudo R.J., Taylor A.M. (2017). Distal axotomy enhances retrograde presynaptic excitability onto injured pyramidal neurons via trans-synaptic signaling. Nat. Commun..

[23] Beaud M., Schmidlin E., Wannier T., Freund P., Bloch J., Mir A., Schwab M.E., Rouiller E.M. (2008). Anti-Nogo-A antibody treatment does not prevent cell body shrinkage in the motor cortex in adult monkeys subjected to unilateral cervical cord lesion. BMC Neurosci..

[24] Onifer S.M., Smith G.M., Fouad K. (2011). Plasticity After Spinal Cord Injury: Relevance to Recovery and Approaches to Facilitate It. Neurotherapeutics.

[25] Dietz V., Fouad K. (2014). Restoration of sensorimotor functions after spinal cord injury. Brain.

[26] Van Middendorp J.J., Goss B., Urquhart S., Atresh S., Williams R.P., Schuetz M. (2011). Diagnosis and Prognosis of Traumatic Spinal Cord Injury. Glob. Spine J..

[27] Nardone R., Höller Y., Brigo F., Orioli A., Tezzon F., Schwenker K., Christova M., Golaszewski S., Trinka E. (2015). Descending motor pathways and cortical physiology after spinal cord injury assessed by transcranial magnetic stimulation: A systematic review. Brain Res..

[28] Nardone R., Langthaler P.B., Höller Y., Bathke A., Frey V.N., Brigo F., Trinka E. (2015). Modulation of non-painful phantom sensation in subjects with spinal cord injury by means of rTMS. Brain Res. Bull..

[29] Yuemin D., Abba K.J., Weihong P. (2005). Neural plasticity after spinal cord injury. Neural Regen. Res..

[30] Xerri C. (2012). Plasticity of cortical maps: Multiple triggers for adaptive reorganization following brain damage and spinal cord injury. Neuroscientist.

[31] Nishimura Y., Isa T. (2009). Compensatory changes at the cerebral cortical level after spinal cord injury. Neuroscientist.

[32] Orlando C., Raineteau O. (2015). Integrity of cortical perineuronal nets influences corticospinal tract plasticity after spinal cord injury. Brain Struct. Funct..

[33] Nardone R., Höller Y., Brigo F., Seidl M., Christova M., Bergmann J., Golaszewski S., Trinka E. (2013). Functional brain reorganization after spinal cord injury: Systematic review of animal and human studies. Brain Res..

[34] Jain N., Florence S.L., Kaas J.H. (1998). Reorganization of somatosensory cortex after nerve and spinal cord injury. News Physiol. Sci..

[35] Hasbargen T., Ahmed M.M., Miranpuri G., Li L., Kahle K.T., Resnick D., Sun D. (2010). Role of NKCC1 and KCC2 in the development of chronic neuropathic pain following spinal cord injury. Ann. N. Y. Acad. Sci..

[36] Venugopal S., Hamm T.M., Crook S.M., Jung R. (2011). Modulation of inhibitory strength and kinetics facilitates regulation of persistent inward currents and motoneuron excitability following spinal cord injury. J. Neurophysiol..

[37] Lu Y., Zheng J., Xiong L., Zimmermann M., Yang J. (2008). Spinal cord injury-induced attenuation of GABAergic inhibition in spinal dorsal horn circuits is associated with down-regulation of the chloride transporter KCC2 in rat. J. Physiol..

[38] Bütefisch C.M. (2004). Plasticity in the Human Cerebral Cortex: Lessons from the Normal Brain and from Stroke. Neuroscientist.

[39] Manganotti P., Acler M., Zanette G.P., Smania N., Fiaschi A. (2008). Motor cortical disinhibition during early and late recovery after stroke. Neurorehabil. Neural Repair.

[40] Brasil-Neto J.P., Cohen L.G., Pascual-Leone A., Jabir F.K., Wall R.T., Hallett M. (1992). Rapid reversible modulation of human motor outputs after transient deafferentation of the forearm: A study with transcranial magnetic stimulation. Neurology.

[41] Cohen L.G., Bandinelli S., Findley T.W., Hallett M. (1991). Motor reorganization after upper limb amputation in man: A study with focal magnetic stimulation. Brain.

[42] Candido Santos L., Gushken F., Gadotti G.M., de Dias B.F., Marinelli Pedrini S., Barreto M.E.S.F., Zippo E., Pinto C.B., de Piza P.V.T., Fregni F. (2020). Intracortical Inhibition in the Affected Hemisphere in Limb Amputation. Front. Neurol..

[43] Tang S.C., Lee L.J.H., Jeng J.S., Hsieh S.T., Chiang M.C., Yeh S.J., Hsueh H.W., Chao C.C. (2019). Pathophysiology of central poststroke pain motor cortex disinhibition and its clinical and sensory correlates. Stroke.

[44] Saturno E., Bonato C., Miniussi C., Lazzaro V., Callea L. (2008). Motor cortex changes in spinal cord injury: A TMS study. Neurol. Res..

[45] Klomjai W., Katz R., Lackmy-Vallée A. (2015). Basic principles of transcranial magnetic stimulation (TMS) and repetitive TMS (rTMS). Ann. Phys. Rehabil. Med..

[46] Farzan F., Vernet M., Shafi M.M.D., Rotenberg A., Daskalakis Z.J., Pascual-Leone A. (2016). Characterizing and modulating brain circuitry through transcranial magnetic stimulation combined with electroencephalography. Front. Neural Circuits.

[47] Sack A.T., Linden D.E.J. (2003). Combining transcranial magnetic stimulation and functional imaging in cognitive brain research: Possibilities and limitations. Brain Res. Rev..

[48] Conde V., Tomasevic L., Akopian I., Stanek K., Saturnino G.B., Thielscher A., Bergmann T.O., Siebner H.R. (2019). The non-transcranial TMS-evoked potential is an inherent source of ambiguity in TMS-EEG studies. Neuroimage.

[49] Potter-Baker K.A., Janini D.P., Frost F.S., Chabra P., Varnerin N., Cunningham D.A., Sankarasubramanian V., Plow E.B. (2016). Reliability of TMS metrics in patients with chronic incomplete spinal cord injury. Spinal Cord.

[50] Lotze M., Laubis-Herrmann U., Topka H. (2006). Combination of TMS and fMRI reveals a specific pattern of reorganization in M1 in patients after complete spinal cord injury. Restor. Neurol. Neurosci..

[51] Petersen J.A., Spiess M., Curt A., Dietz V., Schubert M.N. (2012). Spinal cord injury: One-year evolution of motor-evoked potentials and recovery of leg motor function in 255 patients. Neurorehabil. Neural Repair.

[52] Han D.-S., Li C.-M., Chang C. (2008). Reorganization of the cortico-spinal pathway in patients with chronic complete thoracic spinal cord injury: A study of motor evoked potentials. Acta Derm. Venereol..

[53] Tazoe T., Perez M.A. (2015). Effects of repetitive transcranial magnetic stimulation on recovery of function after spinal cord injury. Arch. Phys. Med. Rehabil..

[54] Yılmaz B., Kesikburun S., Yaşar E., Tan A.K. (2014). The effect of repetitive transcranial magnetic stimulation on refractory neuropathic pain in spinal cord injury. J. Spinal Cord Med..

[55] Ellaway P.H., Vásquez N., Craggs M. (2014). Induction of central nervous system plasticity by repetitive transcranial magnetic stimulation to promote sensorimotor recovery in incomplete spinal cord injury. Front. Integr. Neurosci..

[56] Alexeeva N., Broton J.G., Calancie B. (1998). Latency of changes in spinal motoneuron excitability evoked by transcranial magnetic brain stimulation in spinal cord injured individuals. Electroencephalogr. Clin. Neurophysiol. Electromyogr. Mot. Control.

[57] Shimizu T., Hino T., Komori T., Hirai S. (2000). Loss of the muscle silent period evoked by transcranial magnetic stimulation of the motor cortex in patients with cervical cord lesions. Neurosci. Lett..

[58] Davey N.J. (1998). Responses of thenar muscles to transcranial magnetic stimulation of the motor cortex in patients with incomplete spinal cord injury. J. Neurol. Neurosurg. Psychiatry.

[59] Ellaway P.H., Catley M., Davey N.J., Kuppuswamy A., Strutton P., Frankel H.L., Jamous A., Savic G. (2007). Review of physiological motor outcome measures in spinal cord injury using transcranial magnetic stimulation and spinal reflexes. J. Rehabil. Res. Dev..

[60] Nardone R., Höller Y., Bathke A.C., Orioli A., Schwenker K., Frey V., Golaszewski S., Brigo F., Trinka E. (2015). Spinal cord injury affects I-wave facilitation in human motor cortex. Brain Res. Bull..

[61] Nardone R., Höller Y., Thomschewski A., Bathke A.C., Ellis A.R., Golaszewski S.M., Brigo F., Trinka E. (2015). Assessment of corticospinal excitability after traumatic spinal cord injury using MEP recruitment curves: A preliminary TMS study. Spinal Cord.

[62] Nardone R., De Blasi P., Höller Y., Taylor A.C., Brigo F., Trinka E. (2016). Effects of theta burst stimulation on referred phantom sensations in patients with spinal cord injury. Neuroreport.

[63] Krause P., Szecsi J., Straube A. (2007). Concerning the article by Lotze et al., 2006: Combination of TMS and fMRI reveals a specific pattern of reorganization in M1 in patients after complete spinal cord injury. Restor. Neurol. Neurosci..

[64] Freund P., Rothwell J., Craggs M., Thompson A.J., Bestmann S. (2011). Corticomotor representation to a human forearm muscle changes following cervical spinal cord injury. Eur. J. Neurosci..

[65] Kriz J., Kozak J., Zedka M. (2012). Primary motor cortex inhibition in spinal cord injuries. Neuroendocrinol. Lett..

[66] Ziemann U. (2013). Pharmaco-Transcranial Magnetic Stimulation Studies of Motor Excitability.

[67] Di Lazzaro V., Oliviero A., Meglio M., Cioni B., Tamburrini G., Tonali P., Rothwell J.C. (2000). Direct demonstration of the effect of lorazepam on the excitability of the human motor cortex. Clin. Neurophysiol..

[68] Di Lazzaro V., Pilato F., Dileone M., Profice P., Oliviero A., Mazzone P., Insola A., Ranieri F., Meglio M., Tonali P.A. (2008). The physiological basis of the effects of intermittent theta burst stimulation of the human motor cortex. J. Physiol..

[69] Cirillo J., Calabro F.J., Perez M.A. (2016). Impaired Organization of Paired-Pulse TMS-Induced I-Waves After Human Spinal Cord Injury. Cereb. Cortex.

[70] Di Lazzaro V., Profice P., Ranieri F., Capone F., Dileone M., Oliviero A., Pilato F. (2012). I-wave origin and modulation. Brain Stimul..

[71] Thickbroom G.W. (2011). A model of the contribution of late I-waves to α-motoneuronal activation: Implications for paired-pulse TMS. Brain Stimul..

[72] Belci M., Catley M., Husain M., Frankel H.L., Davey N.J. (2004). Magnetic brain stimulation can improve clinical outcome in incomplete spinal cord injured patients. Spinal Cord.

[73] Schnitzler A., Benecke R. (1994). The silent period after transcranial magnetic stimulation is of exclusive cortical origin: Evidence from isolated cortical ischemic lesions in man. Neurosci. Lett..

[74] Hallett M. (2000). Transcranial magnetic stimulation and the human brain. Nature.

[75] Kobayashi M., Pascual-Leone A. (2003). Basic principles of magnetic stimulation. Lancet.

[76] Burke D., Hicks R., Gandevia S.C., Stephen J., Woodforth I., Crawford M. (1993). Direct comparison of corticospinal volleys in human subjects to transcranial magnetic and electrical stimulation. J. Physiol..

[77] Nielsen J., Petersen N. (1995). Changes in the effect of magnetic brain stimulation accompanying voluntary dynamic contraction in man. J. Physiol..

[78] Schneider C., Lavoie B.A., Barbeau H., Capaday C. (2004). Timing of cortical excitability changes during the reaction time of movements superimposed on tonic motor activity. J. Appl. Physiol..

[79] Geertsen S.S., Zuur A.T., Nielsen J.B. (2010). Voluntary activation of ankle muscles is accompanied by subcortical facilitation of their antagonists. J. Physiol..

[80] Hou J., Nelson R., Nissim N., Parmer R., Thompson F.J., Bose P. (2014). Effect of combined treadmill training and magnetic stimulation on spasticity and gait impairments after cervical spinal cord injury. J. Neurotrauma.

[81] Moxon K.A., Oliviero A., Aguilar J., Foffani G. (2014). Cortical reorganization after spinal cord injury: Always for good?. Neuroscience.

[82] Roy F.D., Zewdie E.T., Gorassini M.A. (2011). Short-interval intracortical inhibition with incomplete spinal cord injury. Clin. Neurophysiol..

[83] Federico P., Perez M.A. (2017). Altered corticospinal function during movement preparation in humans with spinal cord injury. J. Physiol..

[84] Bunday K.L., Urbin M.A., Perez M.A. (2018). Potentiating paired corticospinal-motoneuronal plasticity after spinal cord injury. Brain Stimul..

[85] Urbin M.A., Ozdemir R.A., Tazoe T., Perez M.A. (2017). Spike-timing-dependent plasticity in lower-limb motoneurons after human spinal cord injury. J. Neurophysiol..

[86] Tolmacheva A., Savolainen S., Kirveskari E., Lioumis P., Kuusela L., Brandstack N., Ylinen A., Mäkelä J.P., Shulga A. (2017). Long-Term Paired Associative Stimulation Enhances Motor Output of the Tetraplegic Hand. J. Neurotrauma.

[87] Weise D., Mann J., Ridding M., Eskandar K., Huss M., Rumpf J.J., Di Lazzaro V., Mazzone P., Ranieri F., Classen J. (2013). Microcircuit mechanisms involved in paired associative stimulation-induced depression of corticospinal excitability. J. Physiol..

[88] Petrosyan H.A., Alessi V., Sisto S.A., Kaufman M., Arvanian V.L. (2017). Transcranial magnetic stimulation (TMS) responses elicited in hindlimb muscles as an assessment of synaptic plasticity in spino-muscular circuitry after chronic spinal cord injury. Neurosci. Lett..

[89] Kokotilo K.J., Eng J.J., Curt A. (2009). Reorganization and preservation of motor control of the brain in spinal cord injury: A systematic review. J. Neurotrauma.

[90] Wagle-Shukla A., Ni Z., Gunraj C.A., Bahl N., Chen R. (2009). Effects of short interval intracortical inhibition and intracortical facilitation on short interval intracortical facilitation in human primary motor cortex. J. Physiol..

[91] Gupta S., Jaiswal A., Norman K., DePaul V. (2019). Progressive neuromuscular scoliosis secondary to spinal cord injury in a young patient treated with nonfusion anterior scoliosis correction. Top. Spinal Cord Inj. Rehabil..

[92] Curt A., Van Hedel H.J.A., Klaus D., Dietz V. (2008). Recovery from a spinal cord injury: Significance of compensation, neural plasticity, and repair. J. Neurotrauma.

[93] Rowley N.M., Madsen K.K., Schousboe A., Steve White H. (2012). Glutamate and GABA synthesis, release, transport and metabolism as targets for seizure control. Neurochem. Int..

[94] Kann O. (2016). The interneuron energy hypothesis: Implications for brain disease. Neurobiol. Dis..

[95] Azbill R.D., Mu X., Bruce-Keller A.J., Mattson M.P., Springer J.E. (1997). Impaired mitochondrial function, oxidative stress and altered antioxidant enzyme activities following traumatic spinal cord injury. Brain Res..

[96] Phillips A.A., Ainslie P.N., Krassioukov A.V., Warburton D.E.R. (2013). Regulation of cerebral blood flow after spinal cord injury. J. Neurotrauma.

[97] Melloni L., Molina C., Pena M., Torres D., Singer W., Rodriguez E. (2007). Synchronization of neural activity across cortical areas correlates with conscious perception. J. Neurosci..

[98] Uhlhaas P.J., Singer W. (2010). Abnormal neural oscillations and synchrony in schizophrenia. Nat. Rev. Neurosci..

[99] Craig A., Guest R., Tran Y., Middleton J. (2017). Cognitive Impairment and Mood States after Spinal Cord Injury. J. Neurotrauma.

[100] Wu J., Zhao Z., Sabirzhanov B., Stoica B.A., Kumar A., Luo T., Skovira J., Faden A.I. (2014). Spinal cord injury causes brain inflammation associated with cognitive and affective changes: Role of cell cycle pathways. J. Neurosci..

[101] Gensel J.C., Zhang B. (2015). Macrophage activation and its role in repair and pathology after spinal cord injury. Brain Res..

[102] Ren Y., Young W. (2013). Managing inflammation after spinal cord injury through manipulation of macrophage function. Neural Plast..

[103] Pineau I., Steve L. (2007). Proinflammatory Cytokine Synthesis in the Injured Mouse Spinal Cord: Multiphasic Expression Pattern and Identification of the Cell Types Involved. J. Comp. Neurol..

[104] Zeilhofer H.U. (2008). Loss of glycinergic and GABAergic inhibition in chronic pain-contributions of inflammation and microglia. Int. Immunopharmacol..

[105] Ferrini F., De Koninck Y. (2013). Microglia control neuronal network excitability via BDNF signalling. Neural Plast..

[106] Pozzi D., Rasile M., Corradini I., Matteoli M. (2020). Environmental regulation of the chloride transporter KCC2: Switching inflammation off to switch the GABA on?. Transl. Psychiatry.

[107] Reichelt A.C., Hare D.J., Bussey T.J., Saksida L.M. (2019). Perineuronal Nets: Plasticity, Protection, and Therapeutic Potential. Trends Neurosci..

[108] Carstens K.E., Phillips M.L., Pozzo-Miller L., Weinberg R.J., Dudek S.M. (2016). Perineuronal nets suppress plasticity of excitatory synapses on CA2 pyramidal neurons. J. Neurosci..

[109] Kwok J.C.F., Dick G., Wang D., Fawcett J.W. (2011). Extracellular matrix and perineuronal nets in CNS repair. Dev. Neurobiol..

[110] Casha S., Zygun D., McGowan M.D., Bains I., Yong V.W., John Hurlbert R. (2012). Results of a phase II placebo-controlled randomized trial of minocycline in acute spinal cord injury. Brain.

[111] Rossier J., Bernard A., Cabungcal J.H., Perrenoud Q., Savoye A., Gallopin T., Hawrylycz M., Cuénod M., Do K., Urban A. (2015). Cortical fast-spiking parvalbumin interneurons enwrapped in the perineuronal net express the metallopeptidases Adamts8, Adamts15 and Neprilysin. Mol. Psychiatry.

[112] Härtig W., Derouiche A., Welt K., Brauer K., Grosche J., Mäder M., Reichenbach A., Brückner G. (1999). Cortical neurons immunoreactive for the potassium channel Kv3.1b subunit are predominantly surrounded by perineuronal nets presumed as a buffering system for cations. Brain Res..

[113] Lorenzo Bozzelli P., Alaiyed S., Kim E., Villapol S., Conant K. (2018). Proteolytic Remodeling of Perineuronal Nets: Effects on Synaptic Plasticity and Neuronal Population Dynamics. Neural Plast..

[114] Cabungcal J.H., Steullet P., Morishita H., Kraftsik R., Cuenod M., Hensch T.K., Do K.Q. (2013). Perineuronal nets protect fast-spiking interneurons against oxidative stress. Proc. Natl. Acad. Sci. USA.

[115] Morishita H., Cabungcal J.H., Chen Y., Do K.Q., Hensch T.K. (2015). Prolonged Period of Cortical Plasticity upon Redox Dysregulation in Fast-Spiking Interneurons. Biol. Psychiatry.

[116] Agarwala S., Kalil R.E. (1998). Axotomy-induced neuronal death and reactive astrogliosis in the lateral geniculate nucleus following a lesion of the visual cortex in the rat. J. Comp. Neurol..

[117] Halassa M.M., Fellin T., Haydon P.G. (2007). The tripartite synapse: Roles for gliotransmission in health and disease. Trends Mol. Med..

[118] Le Meur K., Mendizabal-Zubiaga J., Grandes P., Audinat E. (2012). GABA release by hippocampal astrocytes. Front. Comput. Neurosci..

[119] Benedetti B., Matyash V., Kettenmann H. (2011). Astrocytes control GABAergic inhibition of neurons in the mouse barrel cortex. J. Physiol..

[120] Waagepetersen H.S., Sonnewald U., Schousboe A. (2003). Compartmentation of glutamine, glutamate, and GABA metabolism in neurons and astrocytes: Functional implications. Neuroscientist.

[121] Walls A.B., Waagepetersen H.S., Bak L.K., Schousboe A., Sonnewald U. (2015). The Glutamine–Glutamate/GABA Cycle: Function, Regional Differences in Glutamate and GABA Production and Effects of Interference with GABA Metabolism. Neurochem. Res..

[122] MacVicar B.A., Choi H.B. (2017). Astrocytes Provide Metabolic Support for Neuronal Synaptic Function in Response to Extracellular K+. Neurochem. Res..

[123] Hassanzadeh S., Jalessi M., Jameie S.B., Khanmohammadi M., Bagher Z., Namjoo Z., Davachi S.M. (2021). More attention on glial cells to have better recovery after spinal cord injury. Biochem. Biophys. Rep..

[124] Leemhuis E., Giuffrida V., De Martino M.L., Forte G., Pecchinenda A., De Gennaro L., Giannini A.M., Pazzaglia M. (2022). Rethinking the Body in the Brain after Spinal Cord Injury. J. Clin. Med..

[125] Spolidoro M., Sale A., Berardi N., Maffei L. (2009). Plasticity in the adult brain: Lessons from the visual system. Exp. Brain Res..

[126] Bachtiar V., Stagg C.J. (2014). The role of inhibition in human motor cortical plasticity. Neuroscience.

[127] Kolasinski J., Hinson E.L., Divanbeighi Zand A.P., Rizov A., Emir U.E., Stagg C.J. (2019). The dynamics of cortical GABA in human motor learning. J. Physiol..

[128] Castro-Alamancos M., Donoghue J.P., Connors B.W. (1995). Different forms of synaptic plasticity in somatosensory and motor areas of the neocortex. J. Neurosci..

[129] Trepel C., Racine R.J. (2000). GABAergic Modulation of Neocortical Long-Term Potentiation in the Freely Moving Rat. Synapse.

[130] Clarkson A.N., Huang B.S., Macisaac S.E., Mody I., Carmichael S.T. (2010). Reducing excessive GABA-mediated tonic inhibition promotes functional recovery after stroke. Nature.

[131] Murphy S.C., Palmer L.M., Nyffeler T., Müri R.M., Larkum M.E. (2016). Transcranial magnetic stimulation (TMS) inhibits cortical dendrites. eLife.

[132] Nardone R., Höller Y., Thomschewski A., Höller P., Lochner P., Golaszewski S., Brigo F., Trinka E. (2014). Serotonergic transmission after spinal cord injury. J. Neural Transm..

[133] Araneda R., Andrade R. (1991). 5-Hydroxytryptamine2 and 5-hydroxytryptamine1A receptors mediate opposing responses on membrane excitability in rat association cortex. Neuroscience.

[134] Azmitia E.C., Gannon P.J., Kheck N.M., Whitaker-Azmitia P.M. (1996). Cellular localization of the 5-HT(1A) receptor in primate brain neurons and glial cells. Neuropsychopharmacology.

[135] Sheldon P.W., Aghajanian G.K. (1991). Excitatory responses to serotonin (5-HT) in neurons of the rat piriform cortex: Evidence for mediation by 5-HT1C receptors in pyramidal cells and 5-HT2 receptors in interneurons. Synapse.

[136] Sheldon P.W., Aghajanian G.K. (1990). Serotonin (5-HT) induces IPSPs in pyramidal layer cells of rat piriform cortex: Evidence for the involvement of a 5-HT2 -activated interneuron. Brain Res..

[137] Fouad K., Rank M.M., Vavrek R., Murray K.C., Sanelli L., Bennett D.J. (2010). Locomotion after spinal cord injury depends on constitutive activity in serotonin receptors. J. Neurophysiol..

[138] Giménez Y Ribotta M., Provencher J., Feraboli-Lohnherr D., Rossignol S., Privát A., Orsal D. (2000). Activation of locomotion in adult chronic spinal rats is achieved by transplantation of embryonic raphe cells reinnervating a precise lumbar level. J. Neurosci..

[139] Guertin P.A. (2004). Role of NMDA receptor activation in serotonin agonist-induced air-stepping in paraplegic mice. Spinal Cord.

[140] Long J., Federico P., Perez M.A. (2017). A novel cortical target to enhance hand motor output in humans with spinal cord injury. Brain.

[141] Versace V., Langthaler P.B., Höller Y., Frey V.N., Brigo F., Sebastianelli L., Saltuari L., Nardone R. (2018). Abnormal cortical neuroplasticity induced by paired associative stimulation after traumatic spinal cord injury: A preliminary study. Neurosci. Lett..

[142] Kuppuswamy A., Balasubramaniam A.V., Maksimovic R., Mathias C.J., Gall A., Craggs M.D., Ellaway P.H. (2011). Action of 5Hz repetitive transcranial magnetic stimulation on sensory, motor and autonomic function in human spinal cord injury. Clin. Neurophysiol..

[143] Cortes M., Elder J., Rykman A., Murray L., Avedissian M., Stampa A., Thickbroom G.W., Pascual-Leone A., Krebs H.I., Valls-Sole J. (2013). Improved motor performance in chronic spinal cord injury following upper-limb robotic training. NeuroRehabilitation.

[144] Jones E.G. (1993). Gabaergic neurons and their role in cortical plasticity in primates. Cereb. Cortex.

